# Fecal microbial transplantation and a high fiber diet attenuates emphysema development by suppressing inflammation and apoptosis

**DOI:** 10.1038/s12276-020-0469-y

**Published:** 2020-07-17

**Authors:** Yoon Ok Jang, Se Hee Lee, Jong Jin Choi, Do-Hyun Kim, Je-Min Choi, Min-Jong Kang, Yeon-Mok Oh, Young-Jun Park, Yong Shin, Sei Won Lee

**Affiliations:** 1 Department of Pulmonary and Critical Care Medicine, Asan Medical Center, University of Ulsan College of Medicine, Seoul, 05505 Republic of Korea; 2 Department of Convergence Medicine, Asan Medical Institute of Convergence Science and Technology, Asan Medical Center, University of Ulsan College of Medicine, Seoul, 05505 Republic of Korea; 3 Department of Pulmonology, Allergy and Critical Care Medicine, CHA Bundang Medical Center, CHA University, Seongnam-si, 13496 Republic of Korea; 4 Asan Institute for Life Sciences, University of Ulsan College of Medicine, Seoul, 05505 Republic of Korea; 5 Department of Life Science, College of Natural Sciences, Research Institute for Natural Sciences, Research Institute for Convergence of Basic Sciences, Hanyang University, Seoul, 04763 Republic of Korea; 6 Section of Pulmonary, Critical Care and Sleep Medicine, Department of Internal Medicine, Yale University School of Medicine, New Haven, 06520-8057 Connecticut USA; 7 Environmental Disease Research Center, Korea Research Institute of Bioscience and Biotechnology, Daejeon, 34141 Republic of Korea

**Keywords:** Respiratory tract diseases, Translational research

## Abstract

Recent work has suggested a microbial dysbiosis association between the lung and gut in respiratory diseases. Here, we demonstrated that gut microbiome modulation attenuated emphysema development. To modulate the gut microbiome, fecal microbiota transplantation (FMT) and diet modification were adopted in mice exposed to smoking and poly I:C for the emphysema model. We analyzed the severity of emphysema by the mean linear intercept (MLI) and apoptosis by the fluorescent TUNEL assay. Microbiome analysis was also performed in feces and fecal extracellular vesicles (EVs). The MLI was significantly increased with smoking exposure. FMT or a high-fiber diet (HFD) attenuated the increase. Weight loss, combined with smoking exposure, was not noted in mice with FMT. HFD significantly decreased macrophages and lymphocytes in bronchoalveolar lavage fluid. Furthermore, IL-6 and IFN-γ were decreased in the bronchoalveolar lavage fluid and serum. The TUNEL score was significantly lower in mice with FMT or HFD, suggesting decreased cell apoptosis. In the microbiome analysis, *Bacteroidaceae* and *Lachnospiraceae*, which are alleged to metabolize fiber into short-chain fatty acids (SCFAs), increased at the family level with FMT and HFD. FMT and HFD attenuated emphysema development via local and systemic inhibition of inflammation and changes in gut microbiota composition, which could provide a new paradigm in COPD treatment.

## Lung disease: Fecal transplants and fiber reduce airway inflammation

Fecal transplants and high-fiber diets help to mitigate the lung damage caused by smoking in mice, a finding that could lead to new therapeutic interventions for people suffering from emphysema. A team led by Sei Won Lee and Yong Shin from the University of Ulsan College of Medicine, Asan Medical Center, Seoul, South Korea, exposed mice to cigarette smoke and a molecule stimulating the immune system to induce chronic lung disease. The researchers then treated the mice with feces from healthy donor animals or fed them a high-fiber diet. Both interventions altered the microbial community in the gut, leading to increased ability to convert dietary fiber into short chaing fatty acid with immune-modulating effects. Consequently, the mice exhibited less inflammation throughout their bodies, including in the lungs, where alveolar structure was protected from destruction.

## Introduction

Chronic obstructive pulmonary disease (COPD) is a chronic progressive disease with significant worldwide morbidity and mortality^1,2^. Despite progress in management, the overall therapeutic strategy has not changed in recent decades; smoking cessation and bronchodilators are still the main treatment. Pulmonary and systematic inflammation persists in patients with established COPD even after smoking cessation^3^. Bronchodilators improve symptoms, lung function, and quality of life, but they hardly normalize pulmonary function in most cases, and their ability to control inflammation is limited. Therefore, new COPD treatment modalities are needed.

Although cigarette smoking (CS) is the most important risk factor for COPD, only some smokers progress to COPD, suggesting that there are individual differences in CS susceptibility^4,5^. The maintenance of immune homeostasis may be a critical contributor to the susceptibility of a smoker, and this could be due to interactions between the host immune system and microbes^6,7^. Recently, the intimate interactions between gut microbes and the lung were called the gut–lung axis, and microbial dysbiosis in this axis is related to chronic respiratory diseases^8–10^, relatively well described in allergic airway diseases^11,12^. Modulation of gut microbial dysbiosis using diet (prebiotics) and microbe metabolites (postbiotics) demonstrated a beneficial role in the asthma model^12^. Accumulating evidence supports the diverse role of dietary metabolites from microbes in host immune homeostasis^13–15^.

Currently, the functional role of the gut–lung microbial axis in COPD pathogenesis remains poorly understood. Moreover, the potential impact of its modulation as a novel therapeutic strategy for COPD has not been adequately addressed. Here, we used a murine emphysema model that has been widely used for COPD research^16,17^ to demonstrate that gut microbiome modulation would attenuate the development of emphysema.

## Materials and methods

### Emphysema mouse model

Eight-week-old inbred female C57BL/6 mice (Orient Bio, Seongnam, Republic of Korea) were maintained at room temperature (25 °C) with a 12-/12-h light/dark cycle. The mice were exposed to cigarette smoke 5 days/week for 4 weeks with 50 μg (1 μg/μL) of poly(I:C) administration via nasal aspiration twice a week at 3 and 4 weeks^18^. Cigarette smoke exposure was performed using 12 commercial cigarettes per day (4 cigarettes/session, 3 sessions/day, 8.0 mg of tar/cigarette, and 0.70 mg of nicotine/cigarette, Camel) according to a previously described protocol with modifications^18,19^. Control animals inhaled only clean-room air in the cages. This study complied with the recommendations in the Eighth edition of the Guide for the Care and Use of Laboratory Animals. The experimental protocols were approved by the Institutional Animal Care and Use Committee of Asan Medical Center, Seoul, Republic of Korea (2018-01-0027).

### Experimental design

Each experiment was performed with a 4-week study period (Supplementary Fig. 1).

*Study 1*. The effect of FMT on emphysema development was determined in three groups (*n* = 5 mice/group): control, CS exposure only, and CS exposure with FMT.

*Study 2*. The effect of different dietary supplements on emphysema development was determined in five groups (*n* = 4–6 mice/group): control, CS exposure only, CS exposure with a high-fat diet, CS exposure with a high-protein diet, and CS exposure with a high-fiber diet.

*Study 3*. To further investigate the effect of a high-fiber diet and FMT on the development of emphysema, mice were randomly allocated into five groups (*n* = 6 mice/group): control, CS exposure only, CS exposure with a high-fiber diet, CS exposure with FMT, and CS exposure with a high-fiber diet and FMT.

*Study 4*. The effect of short-chain fatty acids (SCFAs) on emphysema development was determined in three groups (*n* = 5 mice/group): control, CS exposure only, and CS exposure with SCFAs.

### Diet modifications

Mice were fed ad libitum with diets based on the purified AIN-76-A diet supplied by Daehan Biolink Co., Ltd. (Chungbuk, Korea). The AIN 76-A diet was modified to study the effect of different diets. The high-protein diet (40% protein) was modified with the increment of casein, high-fat diet (40% fat) with corn oil, high-fiber diet (20% fiber) with 20% cellulose (Study 2), and high-fiber diet (20% fiber) with 10% cellulose and 10% pectin (Study 3).

### Fecal microbiota transplantation

For FMT, 200 mg of fresh feces was collected from control mice or mice receiving the high-fiber diet immediately after defecation before being resuspended in 5 mL of PBS. Homogenates were passed through 40-µm pore-size nylon filters to remove large particulate and fibrous matter. The suspension was centrifuged for 2 min and immediately transplanted into recipient mice using oral gavage with 200 μL of resuspended feces twice a week at 3 and 4 weeks.

### Short-chain fatty acid administration

The SCFAs acetate, propionate, and butyrate (Sigma-Aldrich, St. Louis, MO, USA) were administered in drinking water at 76, 29, and 45 mM, respectively, for the latter 3 weeks of the experiment period.

### Separation and preparation of samples

After 4 weeks, the animals were anesthetized using isoflurane inhalation, and blood samples were collected by heart puncture. Spleens were harvested after acquiring blood from the right atrium. The trachea was catheterized and perfused with 1.5 mL of PBS. The cellular and liquid fractions of BALF were separated by centrifugation at 2200 rpm for 5 min at 4 °C. The cell pellet was suspended in PBS, seeded onto a slide, and stained with Diff-Quick (Sysmex, Kobe, Japan). After ligating the right main bronchus, the left lung lobe was inflated with 0.5% low-melting-point agarose at a constant pressure of 15 cm H_2_O. The left lung lobe was sectioned and fixed in 10% formalin for histological examination. All specimens were collected, fixed, immediately frozen, and stored at −80 °C for analysis.

### Histomorphological assessment

Sections (5 μm) of paraffin-embedded lung lobes were prepared and stained with hematoxylin and eosin (H&E). Emphysematous changes were assessed by measuring the MLI, which is a measurement of the mean interalveolar septal wall distance determined by the number of interruptions in 1-mm lines of the alveolar wall. Four lines were drawn in each field, and at least five random fields were examined per mouse.

### Cytokine-level quantification

IFN-γ and IL-6 levels in the serum and BALF were measured using a commercially available ELISA kit (R&D Systems, Minneapolis, MN, USA) according to the manufacturer’s instructions.

### Quantitative real-time PCR analysis

Total RNA was extracted from liver tissue using TRIzol reagent (Thermo Fisher Scientific, Waltham, MA, USA) according to the manufacturer’s instructions. cDNA was synthesized from total RNA (1 μg) using SSIV VILO Master Mix (Thermo Fisher Scientific). Transcript levels were measured using real-time polymerase chain reaction (PCR) with sequence-specific primers for TNF-α, TGF-β, IL-18, IL-8, MMP-12, MMP-9, IRF-5, Cathepsin S, and IFN-γ (Supplementary Table 1). Amplification reactions were performed with SYBR Green PCR Master Mix (Applied Biosystems, Foster City, CA, USA) in an ABI PRISM 7900HT Sequence Detection System (Applied Biosystems) according to the manufacturer’s instructions. Data were analyzed using SDS 2.2.2 software (Applied Biosystems). The expression levels of the target genes were normalized to *actin* as an endogenous control gene. Relative changes were calculated using the equation 2^−∆∆Ct^.

### TUNEL assay

End labeling of the exposed 3′-OH ends of DNA fragments in paraffin-embedded lung tissue was performed using the terminal deoxynucleotidyl transferase dUTP nick end labeling (TUNEL) In Situ Cell Death Detection Kit (Roche Diagnostics) according to the manufacturer’s instructions. Cell nuclei were counterstained with VECTASHIELD mounting medium containing 4′,6-diamino-2-phenylindole (DAPI) (Vector Laboratories, Burlingame, CA, USA). Fluorescent images were observed under a laser-scanning confocal microscope (LSM-880, Carl Zeiss, Germany). After staining, the number of TUNEL-positive cells (apoptotic cells) was evaluated in ten random fields per mouse. The percentage of TUNEL-positive cells among the total nuclei was computed for each image, and a mean value was obtained for each mouse.

### Fluorescence-assisted cell sorting (FACS) analysis

Lung tissue was chopped and digested using collagenase D (Roche Diagnosis) in Dulbecco’s modified Eagle’s medium (Life Technologies, Carlsbad, CA) for 30 min at 37 °C with agitation. Next, the chopped lung or spleen tissue was passed through a 40-μm cell strainer to obtain single-cell suspensions before RBC lysis. Cells were incubated with LIVE/DEAD Aqua (Thermo Fisher Scientific). Cells were stained with the following monoclonal antibodies: anti-mouse CD45-PerCP-Cy5.5 (BioLegend, San Diego, CA, USA), CD4-PE-Cy7 (BioLegend), CD25-PE (eBioscience), and Foxp3-APC (eBioscience, Waltham, MA, USA). Flow cytometry analysis was performed using a BD FACSCanto II flow cytometer (BD Bioscience, San Jose, CA, USA), and the results were analyzed using FlowJo software (TreeStar, Ashland, OR, USA).

### Quantitative SCFA measurement

Standard metabolites and internal standards were purchased from Sigma-Aldrich. Ten to twenty milligrams of feces was freeze-dried for 12 h using a Benchtop manifold freeze drier and stored at −80 °C with harvested serum until analysis. For sample preparation, the fecal sample was vortexed vigorously with 150 μL of internal standard solution (1 mM propionic acid (C3)-d6) in water and centrifuged at 13,200 rpm for 10 min at 4 °C. The supernatant was then collected. For mouse serum analysis, 20 μL of serum was mixed with 150 μL of internal standard solution (1 mM propionic acid (C3)-d6) in water and mixed well. The solution was centrifuged at 13,200 rpm for 10 min at 4 °C, and the supernatant was collected. Then, 100 μL of 20 mM AABD-SH in dichloromethane, 100 μL of 20 mM TPP in acetonitrile, and 100 μL of 20 mM DPDS in acetonitrile were added to the supernatant. The solution was incubated for 10 min at room temperature while vortexing and dried under vacuum. The dried matter was reconstituted with 20 μL of methanol to prepare for liquid chromatography–tandem mass spectrometry (LC–MS/MS) analysis.

An LC–MS/MS system equipped with 1290 HPLC (Agilent Technologies, Glostrup, Denmark), Qtrap 5500 (ABSciex, Framingham, MA), and a reverse-phase column (Pursuit 5 C18 150 × 2.0 mm, Agilent Technologies) was used. MS was operated in the positive ion mode with a turbo ion-spray voltage of 5500 V using 20 psi curtain gas, 50 psi nebulizer gas, and 50 psi drying gas at 400 °C. The LC separation used mobile phase A (0.1% formic acid in water) and mobile phase B (0.1% formic acid in acetonitrile) and proceeded at 500 µl/min and 40 °C. The separation gradient was as follows: 30% B at 0 min, 50% B for 30 min, 50–30% B for 0.1 min, and 30% B for 4.9 min. Collision energies of 15 V were used for multiple-reaction monitoring (MRM) of each SCFA. LC–MS/MS data were analyzed with Analyst 1.5.2 software (ABSciex).

The extracted ion chromatogram (EIC) corresponding to the specific transition for each metabolite was used for quantitation. The area under the curve of each EIC was normalized to the EIC of the internal standard. The peak area ratio of each metabolite to the internal standard was normalized using the serum volume of a sample before being used for relative comparison. The internal standard for mouse feces was not detected; thus, the results were presented as the analyte peak area.

### Isolation of bacteria-derived EVs and DNA extraction

Bacterial EVs were isolated from the feces using a previously described procedure^20^. Briefly, the fecal samples were filtered through a cell strainer after being diluted in 10 mL of PBS for 24 h. EVs in fecal samples were isolated using differential centrifugation at 10,000 × *g* for 10 min at 4 °C. DNA from EVs was extracted as previously described^21^. Tissue DNA extraction was performed immediately after the sample was cut without boiling or centrifuging. DNA was extracted using a DNA isolation kit (PowerSoil DNA Isolation Kit, MO BIO, USA) following the manufacturer’s instructions. Isolated DNA from each sample was quantified using the QIAxpert system (QIAGEN, Germany).

### Bacterial metagenomic analysis using DNA

Bacterial genomic DNA was amplified with the 16 S_V3_F (5′- TCGTCGGCAGCGTCAGATGTGTATAAGAGACAGCCTACGGGNGGCWGCAG-3′) and 16S_V4_R (5′-GTCTCGTGGGCTCGGAGATGTGTATAAGAGACAGGACTACHVGGGTATCTAATCC-3′) primers specific for the V3–V4 hypervariable regions of the 16S rDNA gene. The libraries were prepared using PCR products according to the MiSeq System guide (Illumina, USA) and quantified using QIAxpert (QIAGEN, Germany). Each amplicon was quantified, and an equimolar ratio was pooled and sequenced on a MiSeq (Illumina, USA) according to the manufacturer’s recommendations.

### Analysis of the bacterial composition in the microbiota

Paired-end reads that matched the adapter sequences were trimmed using cutadapt version 1.1.6^22^. The resulting FASTQ files containing paired-end reads were merged with CASPER and quality-filtered for the Phred (*Q*) score as described by Bokulich^23,24^. A reference-based chimera detection step was conducted to identify the chimeric sequences with VSEARCH against the SILVA gold database^25,26^. Next, the sequence reads were clustered into operational taxonomic units (OTUs) using VSEARCH with a de novo clustering algorithm under a threshold of 97% sequence similarity. The representative OTU sequences were finally classified using the SILVA 128 database with UCLUST (parallel_assign_taxonomy_uclust.py script on QIIME version 1.9.1) under default parameters^27^. The Chao indices, an estimator of the richness of taxa per individual, were estimated to measure the diversity of each sample.

### Statistical analysis

Data were analyzed using the Kruskal–Wallis *H* test, the Mann–Whitney *U* test, and one-way ANOVA, followed by Tukey’s test with SPSS software version 24.0 (IBM, Armonk, NY, USA). All values are expressed as the mean ± standard error (SE). Values of *P* < 0.05 were considered statistically significant.

## Results

### FMT attenuates body weight loss and alveolar destruction in emphysema mice

**Fig. 1:**
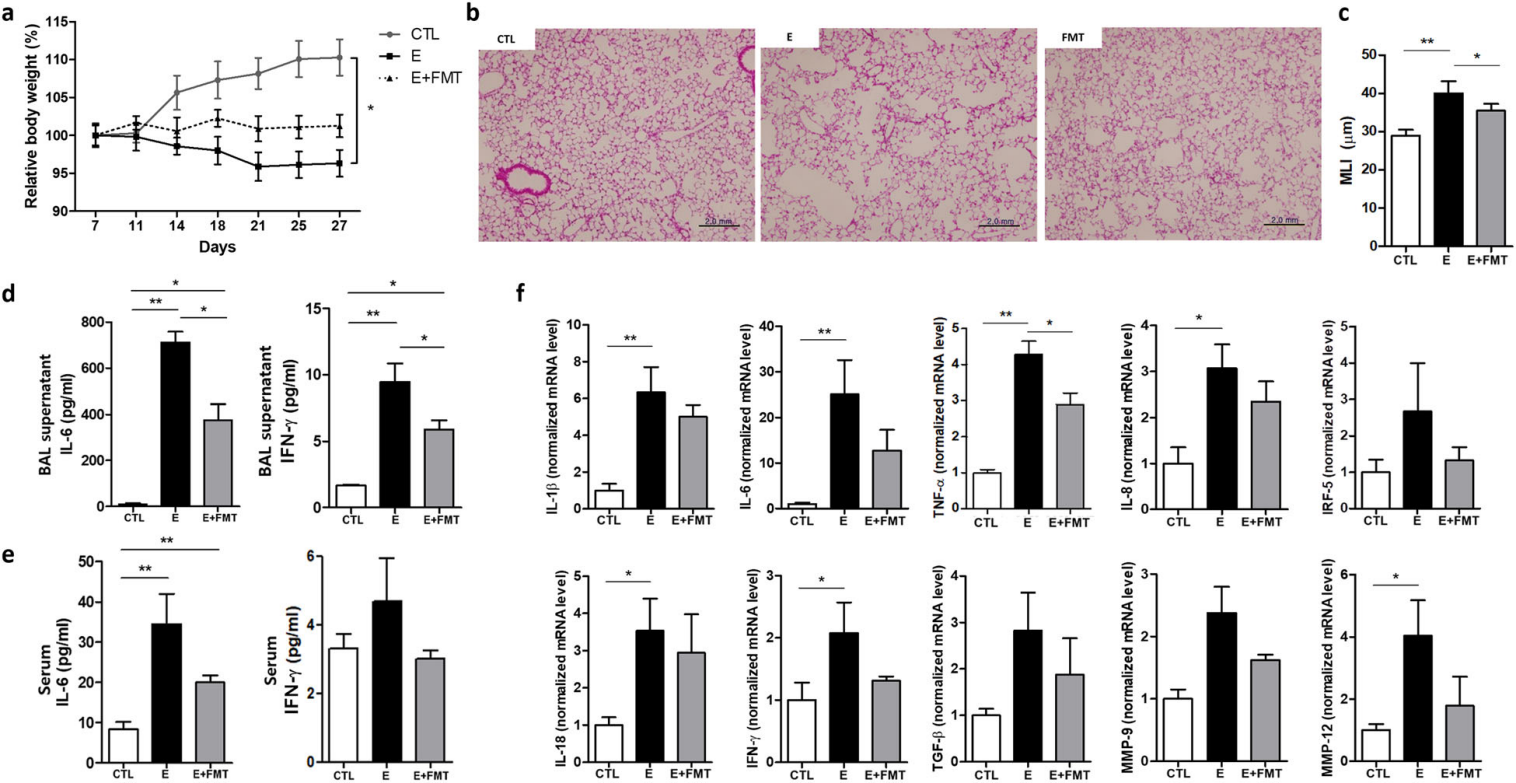
The effect of fecal microbial transplantation on emphysema development (Experiment 1). **a** Relative body weight change during 4 weeks of the experimental period. **b** Representative H&E-stained lung tissues from control, emphysema, and emphysema with FMT mice (magnification: 100×). **c** The mean linear intercept (MLI) of lung tissues from each group. **d, e** The levels of IL-6 and IFN-γ cytokines in the BALF (**d**) and serum (**e**) measured by ELISA. **f** The relative mRNA levels of IL-1β, IL-6, TNF-α, IL-8, IRF-5, IL-18, IFN-γ, TGF-β, MMP-9, and MMP-12 in lung tissues (*n* = 5 mice per group). Values are expressed as the mean ± SE. *P < 0.05 and **P < 0.01. CTL control, E emphysema, FMT fecal microbial transplantation.

To address whether gut microbiota influences emphysema, the first experiment analyzed the effect of FMT on CS-exposure-induced emphysema. Fresh feces from control mice were prepared in a suspension and transplanted into a recipient mouse using oral gavage. The emphysema with the FMT group did not exhibit decreases in relative body weight, but the relative body weight of the emphysema group gradually decreased during the study period compared with the control group (Fig. 1a). CS caused lung parenchymal destruction and airspace enlargement, leading to an increase in the mean linear intercept (MLI), a measurement of the mean interalveolar septal wall distance. Histological analysis of lung tissue sections from each group showed that the alveolar destruction area was greater in the emphysema group than in the control group (Fig. 1b). Importantly, the emphysema with the FMT group showed relatively preserved alveoli compared with the emphysema group (Fig. 1b, c).

In accordance with this, the levels of the cytokines interleukin-6 (IL-6) and interferon-γ (IFN-γ), which were significantly increased in emphysema mouse bronchoalveolar lavage fluid (BALF) and serum compared with the control, were significantly decreased in FMT-treated emphysema compared with the emphysema group (Fig. 1d, e). Moreover, the mRNA expression of other representative proinflammatory mediators, including IL-1β, tumor necrosis factor-α (TNF-α), IL-8, IL-18, and immune modulators, such as interferon regulatory factor-5 (IRF-5) and transforming growth factor-β (TGF-β), was increased in emphysema mice. Importantly, the mRNA levels of IL-1β, TNF-α, IL-8, IRF-5, IL-18, IFN-γ, TGF-β, matrix metalloproteinase-9 (MMP-9), and MMP-12 were decreased in the FMT-treated emphysema group compared with the emphysema group (Fig. 1f). Taken together, these results demonstrate that the transplantation of gut microbiota attenuated the pathological changes in the lungs and influenced the inflammatory response associated with CS-exposure-induced emphysema, suggesting that the transfer of eubiotic feces could counterbalance the local and systemic effects of emphysema.

### High-fiber and high-protein diets have a protective role in emphysema mice

Previous studies reported that diet has a major influence on gut microbiota and immune responses in inflammatory diseases^28–30^. Hence, we investigated whether dietary modification could affect the pulmonary inflammatory response in mice with CS-exposure-induced emphysema. Mice were fed a high-fat diet (40% fat), a high-protein diet (40% protein), or a high-fiber diet (20% fiber). The detailed composition of each diet is described in the “Materials and methods” section.

**Fig. 2:**
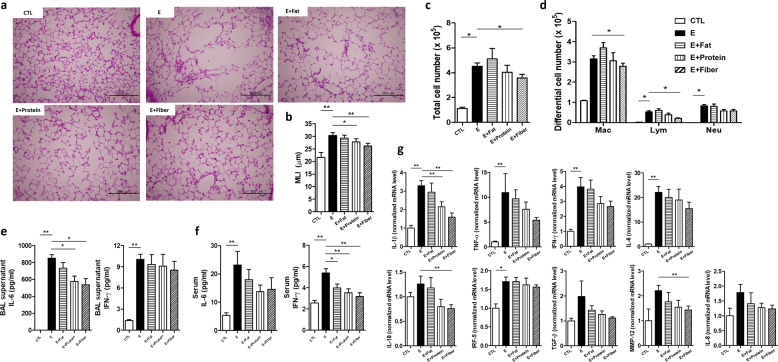
Dietary modification alters inflammation and the degree of alveolar destruction (Experiment 2). **a** Representative H&E-stained lung tissues from mice in the control, emphysema, emphysema with high-fat diet, emphysema with high-protein diet, and emphysema with high-fiber diet groups (magnification: 200×). **b** The MLI of lung tissues from each group. **c** Total number of cells in the BALF infiltrating the airways. **d** Differential cell numbers of BALF in each group. **e, f** The levels of the cytokines IL-6 and IFN-γ in the BALF (**e**) and serum (**f**) measured using ELISA. **g** The relative mRNA levels of IL-1β, TNF-α, IFN-γ, IL-6, IL-8, IL-18, IRF-5, TGF-β, and MMP-12 in lung tissues (*n* = 4 control mice, *n* = 6 emphysema mice, *n* = 6 emphysema with high-fat diet mice, *n* = 6 emphysema with high-protein diet mice, and *n* = 6 emphysema with high-fiber diet mice). Values are expressed as the mean ± SE. *P < 0.05 and **P < 0.01. CTL control, E emphysema.

Histological analysis showed that lung alveolar destruction was the most severe in the emphysema group (Fig. 2a). Mice receiving a high-fiber diet or high-protein diet presented less severe forms of histological emphysema compared with the emphysema group (Fig. 2a). This observation was further confirmed by the MLI measurement, which was significantly lower in mice receiving high-fiber or high-protein diets compared with emphysema mice (Fig. 2b). These results showed that a high-fiber or high-protein diet attenuated CS-exposure-induced emphysema. Above all, the high-fiber diet was more preventive against emphysema than the high-protein diet.

Inflammatory cell infiltration was increased in the BALF of all CS-exposed groups (Fig. 2c). This inflammation was characterized by increased macrophage infiltration, which was the highest in the emphysema group fed a high-fat diet. The number of macrophages and lymphocytes in BALF was significantly lower in mice fed a high-fiber diet than in emphysema mice (Fig. 2d).

The levels of the cytokines IL-6 and IFN-γ were increased in the BALF and serum of emphysema mice, presumably in association with increased macrophage infiltration^31^. IL-6 and IFN-γ levels in BALF were the lowest in the high-fiber diet group (Fig. 2e). Moreover, IL-6 and IFN-γ serum levels decreased in mice receiving modified diets (Fig. 2f). The relative mRNA levels of IL-1β, TNF-α, IFN-γ, IL-6, IL-8, IL-18, IRF-5, TGF-β, and MMP-12 were the lowest in the mice fed a high-fiber diet compared with other CS-exposed mice (Fig. 2g). Here, we showed that a high-fiber diet attenuates the overall magnitude of the inflammatory response, protecting against CS-exposure-induced emphysema development. Moreover, these results indicate that a high-fiber diet may confer immune-modulating effects derived from the by-products of dietary fiber.

### A combination of FMT and a high-fiber diet further reduces inflammation

Both probiotics and prebiotics influence gut microbiota composition. The next experiment was performed to address the combined effect of FMT and a high-fiber diet on gut microbiota and emphysema. Fresh feces from mice fed a high-fiber diet were prepared for FMT and transplanted into recipient mice using oral gavage.

**Fig. 3:**
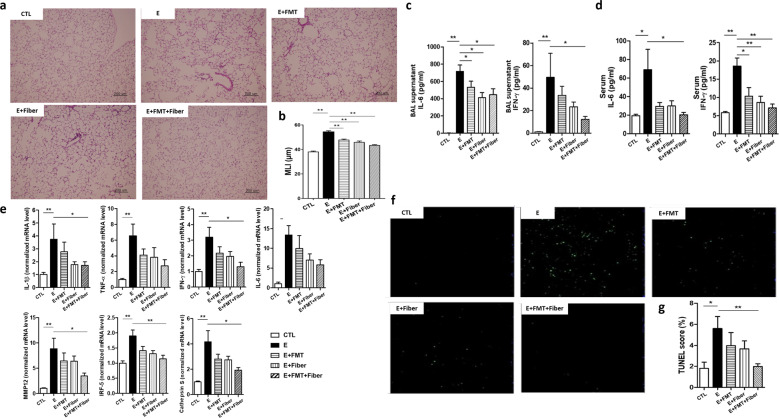
The effect of FMT and dietary modification on emphysema (Experiment 3). **a** Representative H&E-stained lung tissues from mice in the control, emphysema, emphysema with FMT, emphysema with high-fiber diet, and emphysema with both FMT and high-fiber diet groups (magnification: 100×). **b** The MLI of lung tissues from each group. **c, d** The levels of IL-6 and IFN-γ cytokines in the BALF (**c**) and serum (**d**) measured using ELISA. **e** The relative mRNA levels of IL-1β, TNF-α, IFN-γ, IL-6, MMP-12, IRF-5, and Cathepsin S in the lung tissue. **f** Representative TUNEL images of lung tissue from mice in the control, emphysema, emphysema with FMT, emphysema with high-fiber diet, and emphysema with both FMT and high-fiber diet groups. **g** TUNEL score (%) of each group. TUNEL score (%) = TUNEL cells × 100/total cells (*n* = 6 mice per group; *n* = 4 control mice for IFN-γ and IL-6 analysis in BALF and serum). Values are expressed as the mean ± SE. *P < 0.05 and **P < 0.01. CTL control, E emphysema, FMT fecal microbial transplantation.

Similar to previous results, alveolar destruction was the most severe in emphysema mice. Alveolar structures were relatively preserved in other CS-exposed mice compared with emphysema mice (Fig. 3a). Gut microbiome modulation using FMT, a high-fiber diet, or both significantly lowered the MLI (Fig. 3b).

As in the above results, IL-6 and IFN-γ cytokine levels in BALF and serum were lower in both the FMT and high-fiber diet groups compared with the emphysema group. A combination of FMT and a high-fiber diet further reduced IL-6 and IFN-γ levels in both BALF and serum (Fig. 3c, d). The relative mRNA levels of IL-1β, TNF-α, IFN-γ, IL-6, IRF-5, MMP-12, and Cathepsin S were the lowest in mice fed both FMT and a high-fiber diet (Fig. 3e). In accordance with our previous results, we confirmed that FMT and a high-fiber diet attenuated CS-exposure-induced emphysema. Moreover, this study indicated that combined FMT and a high-fiber diet had more potent preventive effects on the development of emphysema than FMT or a high-fiber diet.

We next determined whether CS exposure influences cell apoptosis, and whether a combination of FMT and a high-fiber diet would affect the pathogenesis of this cell death response. Cell apoptosis was the most prominent in emphysema mice (Fig. 3f), and it was significantly lower in both the FMT and high-fiber diet groups than in the emphysema group (Fig. 3g).

Since regulatory T cells (T_regs_) are important for immune homeostasis, we hypothesized that T_regs_ may play a role in immune response attenuation. We examined the production of CD4^+^CD25^+^Foxp3^+^ T cells in the spleen (Supplementary Fig. 2a, Fig. 2b). The mice fed FMT and a high-fiber diet showed reduced production of CD4^+^CD25^+^Foxp3^+^ T cells compared with emphysema mice (Supplementary Fig. 2b), suggesting a correlation between inflammatory symptoms and T_reg_ cell number.

These results demonstrated that gut modulation using both FMT and a high-fiber diet had additive effects in attenuating both local and systemic inflammation, thereby attenuating emphysema development.

### FMT and dietary modification alter the gut microbiota and local SCFA concentration

The above results enabled us to hypothesize that the beneficial effects of FMT combined with a high-fiber diet would induce a shift in microbial composition, resulting in changes to gut microbiota-derived metabolites. SCFAs have been extensively investigated among various metabolites for their beneficial immunomodulatory role^15,32^. To verify this hypothesis, fecal samples from each group in the second and third experiments were retrieved for microbial analysis. Bacterial extracellular vesicles (EVs) were additionally isolated from the feces of mice in the third experiment. We also analyzed representative SCFA concentrations in fecal samples and serum from the third experiment.

**Fig. 4:**
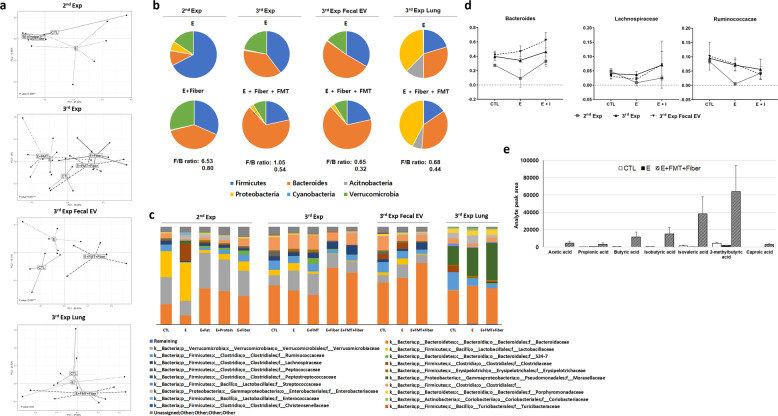
FMT and dietary modification alter the gut microbiota and local SCFA concentration. **a** Principal component analysis (PCA) of fecal samples, fecal exosomes, or lung tissues from different experiments. **b** The proportions of the Firmicutes, Bacteroidetes, Actinobacteria, Proteobacteria, Cyanobacteria, and Verrucomicrobia phyla in emphysema mice compared with mice with diet modifications. **c** Family-level pyrosequencing analysis of the microbial composition of feces, fecal exosomes, or lung tissues from mice treated with FMT, dietary modification, or both. **d** Bacteria-specific relative composition changes throughout all experiments at the family level. Intervention (I) indicated below; 2nd Exp = diet modification, including fat, protein, and fiber diets (*n* = 18); 3rd Exp = FMT, high-fiber diet, or both (*n* = 17); 3rd Exp Fecal EV = FMT and high-fiber diet (*n* = 5). **e** SCFA analysis in the feces. Area ratio = analyte peak area/internal standard peak area (*n* = 4 control mice, *n* = 6 emphysema mice, and *n* = 6 emphysema mice with both FMT and high-fiber diet). CTL control, E emphysema, I intervention.

Principal component analysis (PCA) showed that the control mice, emphysema mice, and emphysema mice with the assigned intervention exhibited distinct microbial community structures in feces and fecal EVs but not in lung tissue (Fig. 4a). FMT and a high-fiber diet were significantly associated with different microbial community structures in the gut. The microbial composition was further analyzed at the phylum and family levels. At the phylum level, Firmicutes and Bacteroides were the major microbiota in feces and fecal EVs (Fig. 4b). Firmicutes species were the most dominant in fecal samples from emphysema mouse feces. FMT and a high-fiber diet led to an increase in the abundance of the Bacteroides phylum, reducing the Firmicutes/Bacteroides (F/B) ratio. In fecal EVs, Bacteroides was the major phylum in both emphysema mice and emphysema mice with FMT and a high-fiber diet. However, the intervention similarly increased the Bacteroides phylum, decreasing the F/B ratio. The decreased F/B ratio in mice treated with FMT and a high-fiber diet was also observed in lung tissue microbiota, where the microbial composition differed from that of the gut microbiota.

Pyrosequencing at the family level showed that emphysema mice generally had increased *Lactobacillus* family members and decreased *Bacteroidaceae* family members compared with control mice. Comparatively, a high-fiber diet and FMT led to a decrease in the *Lactobacillus* family and an increase in the *Bacteroidaceae* family (Fig. 4c). At the bacterial-specific level, the *Bacteroidaceae* family was decreased in emphysema mice and increased in emphysema mice treated with FMT and a high-fiber diet in both fecal samples and fecal EVs. The *Lachnospiraceae* family in the Firmicutes phylum, which allegedly produces SCFAs, including propionate, similar to the *Bacteroidaceae* family, had a similar change in relative abundance in feces and fecal EVs (Fig. 4d). The *Ruminococcus* family, which also metabolizes dietary fiber into SCFAs, showed a similar trend in the fecal sample of the second experiment.

The local concentration of SCFAs was notably higher in emphysema mice fed FMT and a high-fiber diet than in emphysema mice (Fig. 4e).

### Effect of oral SCFA administration on emphysema

Since dietary modification influenced the local SCFA concentration, we directly administered SCFAs. We expected that SCFAs themselves might decrease the local and systemic inflammatory response and attenuate emphysema. The mixture of SCFAs, including acetate, propionate, and butyrate, was administered to emphysema mice by drinking water for the last 3 weeks of the experimental period.

**Fig. 5:**
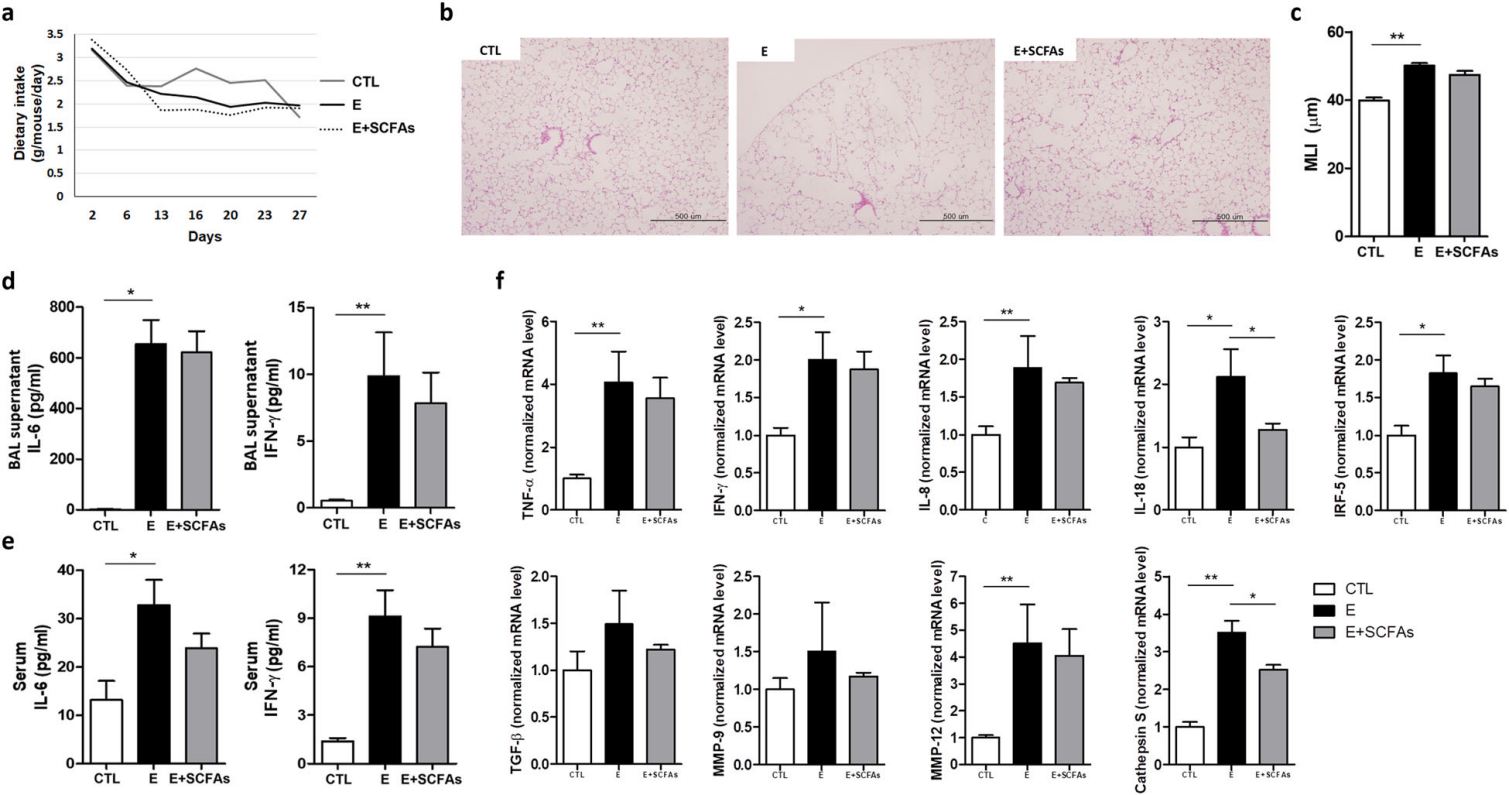
SCFA supplementation decreased the inflammatory response and emphysema (Experiment 4). **a** Dietary intake changes during the 4 weeks of the experimental period. **b** Representative H&E-stained lung tissues from mice in the control, emphysema, and emphysema with SCFA supplementation groups (magnification: 100×). **c** The MLI of lung tissues from each group. **d, e** The levels of the cytokines IL-6 and IFN-γ in the BALF (**d**) and serum (**e**) measured using ELISA. **f** The relative mRNA levels of TNF-α, IFN-γ, IL-8, IL-18, IRF-5, TGF-β, MMP-9, MMP-12, and Cathepsin S in the lung tissue (*n* = 5 mice per group; *n* = 4 control mice for IL-6 analysis in BALF). Values are expressed as the mean ± SE. *P < 0.05 and **P < 0.01. CTL control, E emphysema.

Dietary intake was generally lower in the CS-exposure group than in the control group (Fig. 5a). The decrease in dietary intake was prominent during the initial 1–2 weeks of CS exposure. The dietary intake was similar between the emphysema mice and the emphysema mice with SCFAs for the third and fourth weeks of the study period.

The emphysema mice showed severe emphysema, but SCFA administration resulted in decreased alveolar destruction (Fig. 5b). Emphysema mice receiving SCFAs had a lower MLI than emphysema mice (Fig. 5c). IL-6 and IFN-γ cytokine levels in the BALF and serum decreased in emphysema mice receiving SCFAs compared with emphysema mice (Fig. 5d, e).

The relative mRNA levels of TNF-α, IFN-γ, IL-8, IL-18, IRF-5, and TGF-β increased in the emphysema mice. Thus, the expression of factors associated with tissue destruction and healing, including MMP-12, MMP-9, and Cathepsin S, was also increased in emphysema mice. In contrast, emphysema mice with SCFAs showed reduced expression of the noted mRNAs compared with emphysema mice (Fig. 5f). Among them, IL-18 and Cathepsin S expression levels were significantly altered in response to SCFA administration. We observed that postbiotic SCFA administration attenuated emphysema development, but SCFAs were less effective than a prebiotic high-fiber diet. These results indicate that a prebiotic high-fiber diet has more potent preventive effects, and is associated with better lung function related to emphysema development.

## Discussion

Here, we demonstrated the therapeutic potential of FMT and a high-fiber diet in emphysema treatment. The modulation of gut microbiota with prebiotics and FMT changed the gut microbiota composition, consequently attenuating smoking-induced emphysema. Prebiotics and a high-fiber diet showed the most prominent benefit among various diets. Microbiota transplantation with feces from a high-fiber diet further improved emphysema. The therapeutic implication of gut microbiota has been mainly studied in gastrointestinal diseases, and it has not been tried in pulmonary diseases, except for a few allergic diseases. This study suggested the meaningful possibility of a new emphysema treatment, offering hope to treat a chronic incurable disease.

Extensive studies have revealed that the gut microbiota is crucial in maintaining host homeostasis and health via immune system interactions^6,33^. The gut microbiota has a systemic impact beyond the intestine via various metabolites^34,35^. The lungs are no exception: accumulating data support a link between gut microbial dysbiosis and chronic respiratory disease^8–10,29^. Low gut microbial diversity is associated with an increased risk of allergic disease development during childhood^36^. Children with cystic fibrosis also display low gut microbial diversity with distinct changes in microbial composition^37^. Moreover, previous studies have reported that EVs play a pivotal role in COPD, and gut microbiota and microbiota-derived EVs influence the lung microenvironment^38–40^. Although a number of studies have demonstrated dysbiotic airway microbiota in patients with COPD, including overall increases in Firmicutes and Proteobacteria and a decrease in Bacteroidetes^8^, the role of intestinal microbiota in COPD development and progression is less clear.

The gut microbiota is influenced by several factors, and diet is considered a key factor that regulates the microbial composition and its metabolic function^28,30,41^. Therefore, it is natural to expect that a healthy diet or direct transfer of good microbiota would have a beneficial effect on the lung, especially under pathological conditions. Among various diets, a high-fiber diet has been extensively studied for its beneficial effect on the “diet–microbiota–immunity” link^15,29,30^. Based on these lines of evidence, we investigated whether EVs secreted by the gut microbiota under a high-fiber diet played a role in emphysema, considering that bacterial EVs contribute to inflammation in the lung microenvironment. In this study, a high-fiber diet was significantly associated with attenuation of both local and systemic inflammation, alveolar destruction, and cellular apoptosis. Dietary modification with a high-fiber diet led to a distinct change in the gut microbial structure, including a decrease in the F/B ratio and an increase in local SCFA concentrations. The combination of FMT and a high-fiber diet additionally counterbalanced the harmful effects of CS exposure. The beneficial effects of the high-fiber diet in decreasing systemic inflammation were previously reported^42–44^. Inverse associations were noted between dietary fiber intake and the activity of serum C-reactive protein (CRP), a marker of acute inflammation^42,43^. A small randomized crossover trial demonstrated that a high-fiber diet decreases CRP levels by 20–30% from baseline^44^. The relationship between dietary fiber intake and lung function was evaluated in prospective cohort studies in the United Kingdom and United States^45–47^. In those studies, fiber-rich diets were positively associated with improved lung function and negatively associated with the risk of COPD development. In large epidemiologic studies^48,49^, a high-fiber diet was also significantly associated with reduced respiratory-related deaths.

The underlying mechanism through which a high-fiber diet plays a beneficial role in lung health with a “diet–microbiota–immunity” link has been researched extensively^15,50^. SCFAs are considered one of the key microbial metabolites in this link. In the allergic airway model, a fiber-rich diet changes the gut microbiota and affects the lung immune response by increasing SCFAs^12,51,52^. Dietary fiber-derived SCFAs also demonstrated a protective role in tissue damage in the influenza-infected mouse model^53^. SCFAs dampen the harmful innate immune response by activating G-protein receptors, inhibiting histone deacetylase, and serving as energy substrates for many immune-regulating cells^15,29^. Here, the high-fiber diet increased the local SCFA concentration and improved inflammation and alveolar destruction. Meanwhile, a prebiotic high-fiber diet was more effective than postbiotic SCFAs in emphysema. Although SCFAs are known to have beneficial effects on the immune system, the effects of SCFAs in pulmonary diseases and the underlying mechanisms are still elusive. Thus, we considered that the dose and proportion of SCFAs, and the period and route of administration, may influence the results. Further research on the role of SCFAs in emphysema is necessary.

Since the gut microbiota produces other metabolites, such as amino acid derivatives and polyamines, the beneficial effects of the high-fiber diet more likely came from the combination with other metabolites rather than from SCFAs alone. In this context, the result of the recent metabolomic study that suggested the association between several metabolites and clinical outcomes in patients with COPD is not surprising^54^. Further studies with metabolomics may help to disclose potential diagnostic and therapeutic candidates in emphysema.

COPD is primarily considered a respiratory disease, but it also includes systemic manifestations such as weight loss or being underweight, which are poor prognostic factors^55,56^. Therefore, it seems reasonable to hypothesize that COPD prognosis can be improved by managing systemic manifestations such as weight loss^57^. Here, mice with FMT were protected from weight loss during emphysema development. The composition of Bacteroides, known to be associated with obesity^58^, increased when emphysema improved. The proportion of Bacteroidetes (specifically, *Prevotella* spp.) is also known to be significantly decreased in COPD^59^. The intestine is the most densely bacteria-colonized surface of the human body, and the lower respiratory tract is one of the least populated. Several bacteria appear in the intestine before being detected in the respiratory tract^60^. This points toward a contribution of gut microbes and their therapeutic application for respiratory diseases. It may also be an interesting approach to determine if FMT from diseased donors can affect lung health in further research. It was documented that transferred microbiota from mice with metabolic abnormalities can cause similar systemic changes in recipient mice^61^.

Here, we demonstrated the potential role of gut microbiota modulation in emphysema, and we identified certain families of microbiota presenting consistent changes throughout the experiments. The beneficial effects of probiotics in chronic lung disease were addressed in preclinical studies or small-scale clinical trials. Administration of various probiotic bacteria attenuated the allergic response in a murine model^8,29^. In patients with cystic fibrosis, administration of *Lactobacillus rhamnosus* GG was associated with restored intestinal microbiota and decreased local inflammation^37^. There are no reports on the role of probiotics in COPD progression. Understanding the complex relationship between prebiotics, specific microbiota, and metabolites in emphysema will provide future therapeutic applications that prevent COPD progression.

In conclusion, we demonstrate that FMT and a high-fiber diet modulate gut microbiota and attenuate the degree of emphysema in a murine model. FMT and a high-fiber diet decreased local and systemic inflammation and protected against alveolar destruction and cellular apoptosis. These findings highlight the importance of dietary fiber in patients with COPD, and provide new interventional insights for preventing or delaying COPD progression.

## Supplementary information

Supplementary material
