# Supplementary material for: Fecal microbial transplantation and a high fiber diet attenuates emphysema development by suppressing inflammation and apoptosis
Source: Exp Mol Med. 2020 Jul 17;52(7):1128–39. doi: 10.1038/s12276-020-0469-y (PMC8080776; doi:10.1038/s12276-020-0469-y)
Supplement: Supplementary file 1 — Supplementary material [file 12276_2020_469_MOESM1_ESM.docx]

**Supplementary Data**

**
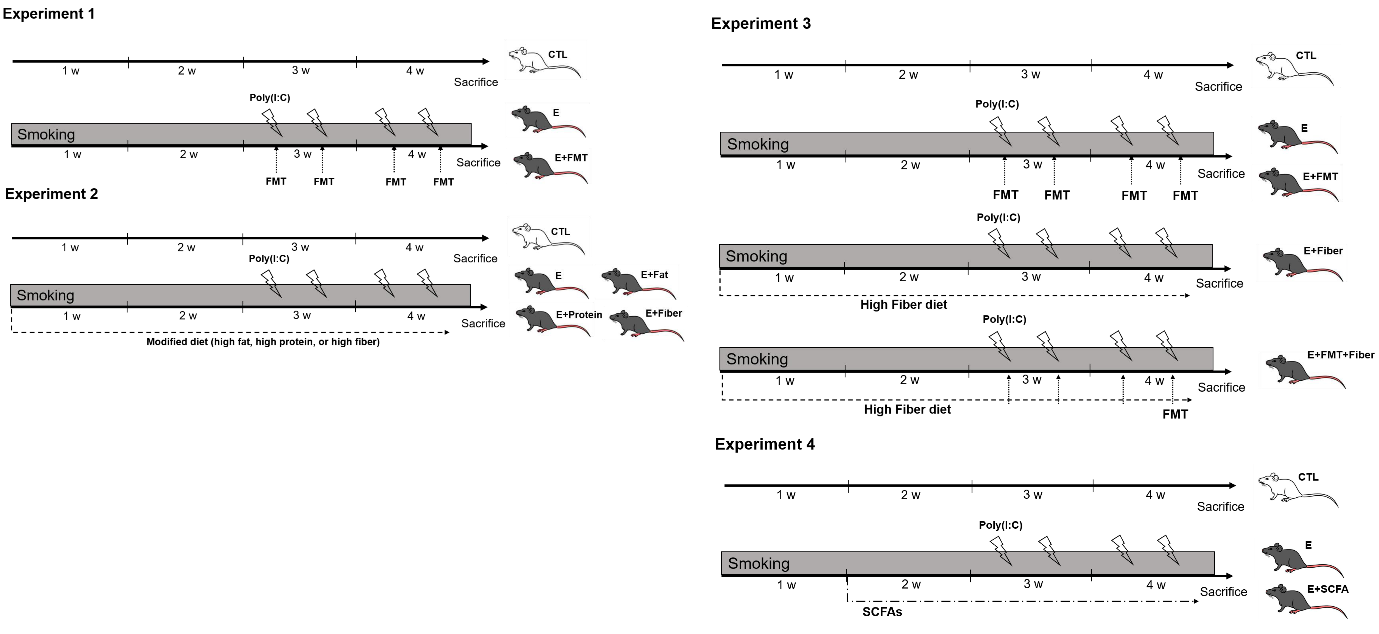
**

**Supplementary Fig. 1 Experimental design.** Experiment 1: FMT study. Experiment 2: Diet study. Experiment 3: FMT and Fiber diet study. Experiment 4: SCFAs study. CTL, control; E, emphysema; FMT, Fecal microbial transplantation; SCFAs, short chain fatty acids.


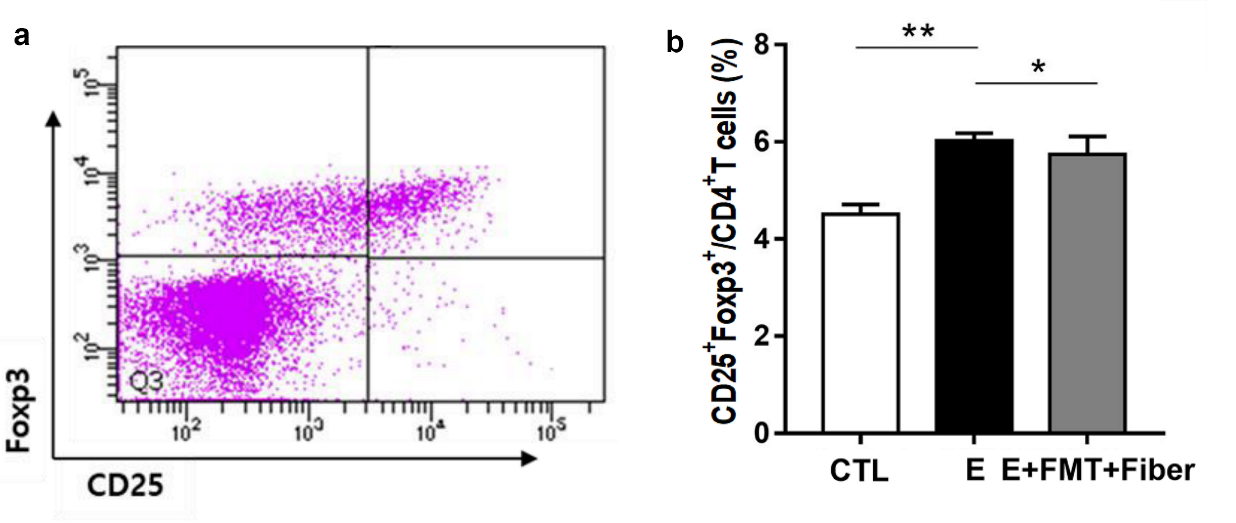


**Supplementary Fig. 2 Fluorescence-assisted Cell Sorting (FACS) analysis. a** Representative gating image are shown. **b** Percentage of CD25+Foxp3+ T cells retrieved from spleens. (n = 6 mice per group; n = 4 control mice. Values are expressed as the mean ± SE. **P* < 0.05 and ***P* < 0.01. CTL = control; E = Emphysema; FMT = Fecal microbial transplantation.

**Supplementary Table 1**. **Quantitative PCR primer sequences**

| Gene | Forward/reverse | Primer sequence 5′-3′ |
| --- | --- | --- |
| Actin | Forward | \| AAGAGCTATGAGCTGCCTGA \| \| --- \| |
|  | Reverse | \| CACAGGATTCCATACCCAAG \| \| --- \| |
| IFN-γ | Forward | AAGCGTCATTGAATCACACCTG |
|  | Reverse | TGACCTCAAACTTGGCAATACTC |
| IL-1β | Forward | CCAAGCAACGACAAAATACC |
|  | Reverse | GTTGAAGACAAACCGTTTTTCC |
| TGF-β | Forward | CTGCTGACCCCCACTGATAC |
|  | Reverse | GTGAGCGCTGAATCGAAAGC |
| TNF-α | Forward | GACAGTGACCTGGACTGTGG |
|  | Reverse | TGAGACAGAGGCAACCTGAC |
| IL-6 | Forward | TGTGCAATGGCAATTCTGAT |
|  | Reverse | GGTACTCCAGAAGACCAGAGGA |
| IL-8 | Forward | TTGGTGATGCTGGTCATCTT |
|  | Reverse | TTTAGATGCAGCCCAGACAG |
| IL-18 | Forward | GCTGTGACCCTCTCTGTGAA |
|  | Reverse | GGCAAGCAAGAAAGTGTCCT |
| MMP-9 | Forward | CTGGACAGCCAGACACTAAAG |
|  | Reverse | CTCGCGGCAAGTCTTCAGAG |
| MMP-12 | Forward | GGCCATTCCTTGGGGCTGCA |
|  | Reverse | GGGGGTTTCACTGGGGCTCC |
| IRF-5 | Forward | GCTGGCTACAGGGTTCTGAG |
|  | Reverse | CTGCTGGCTTCATTTCTTCC |
| Cathepsin S | Forward | GCCAGCCATTCCTCCTTCTT |
|  | Reverse | AGCCAACCACAAGAACACCA |

IFN-γ, interferon-γ; IL, interleukin; TGF-β, transforming growth factor-β; TNF-α, tumor necrosis factor-α; MMP, matrix metalloproteinase; IRF-5, interferon regulatory factor-5
